# Influence of health promoting lifestyle on health management intentions and behaviors among Chinese residents under the integrated healthcare system

**DOI:** 10.1371/journal.pone.0263004

**Published:** 2022-01-25

**Authors:** Chi Zhou, Weijun Zheng, Fang Tan, Sihong Lai, Qi Yuan

**Affiliations:** 1 School of Public Health, Hangzhou Normal University, Hangzhou, Zhejiang, China; 2 School of Basic Medical Science, Zhejiang Chinese Medical University, Hangzhou, Zhejiang, China; 3 Research Division, Institute of Mental Health, Singapore, Singapore; University of Southern Queensland, AUSTRALIA

## Abstract

**Background:**

Health promoting lifestyle is an important influencing factor of individual health status. This study aims to assess the health promoting lifestyle of community residents in China, and explore its association with their health management intention and behaviors during the integrated healthcare system reform.

**Methods:**

A total of 666 residents were recruited from six county level hospitals and 12 community health centers from July to August 2019 in Zhejiang Province, China. Health promoting lifestyle was measured by the Chinese version Health Promoting Lifestyle Profile-II scale (HPLP-II).

**Results:**

The average total score of HPLP-II among our sample was 130.02±23.19. Among the six domains, interpersonal relationship had the highest average score (2.68±0.50), and physical activity scored the lowest (2.21±0.59). Total score of HPLP-II scale was negatively associated with being male (β = -0.13, p<0.01; Ref: female), positively associated with being students (β = 0.15, p<0.01; Ref: self-employed), and positively associated with a monthly per capita income of more than 8000 RMB (β = 0.15, p<0.01; Ref: less than 3000 RMB). The domain scores of HPLP-II were significantly correlated with residents’ health management intention and their behavior on following doctors’ advice or not.

**Conclusions:**

The health promoting lifestyles of community residents in China are at moderate levels. Improving residents’ healthy lifestyle levels might be helpful for changing their health management intentions or behaviors.

## Introduction

Over the past few decades China has gone through major society and economic developments. The average life expectancy increased from 71.40 to 77.3, and the average household income increased from 12514.2 RMB to 30732.8 RMB from 2000 to 2019 [1, 2]. Together with the great achievements are the huge lifestyle changes among Chinese population and a much higher prevalence of non-communicate diseases and their associated heavy financial burdens [3]. For example, the increasing numbers of people traveling by vehicles have led to significant reductions of physical activity levels among the residents, and as a result increased the rate of overweight, obesity and type 2 diabetes in the urban areas [4–6]. Similarly, diets higher in salt, sugar and fat have gave rise to the greater prevalence of metabolic and cardiovascular diseases [7, 8]. For instance, the prevalence of hypertension, diabetes, and metabolic diseases were 181.4‰, 53.1‰ and 62.5‰ respectively among Chinese residents aged 18 and above in 2018; compared to 26.2‰, 5.6‰, 7.5‰ in 2003 [1, 2]. Among the middle aged and elderly people in the capital city of Zhejiang Province, 38.75%, 29.01%, and 16.05% of residents were found to have hypertension, fasting hyperglycemia (>6.1 mmol/L), and elevated total cholesterol (≥6.22 mmol/L) in 2018; compared to 27.04%, 22.65%, and 7.96% in 2010 [9]. Healthy lifestyle plays an important role on individual’s health. Studies suggested that healthy diet, physical activity, healthy body weight, alcohol and tobacco control were associated with lower prevalence of obesity, diabetes, cardiovascular diseases, and cancers [10–12]. This in turn could constraint the costs on health care, especially among aging societies [13, 14].

The Health-promoting lifestyle profile-II (HPLP-II) is an international instrument widely used to measure individuals’ healthy lifestyles, and it has six domains including health responsibility, physical activity, nutrition, spiritual growth, interpersonal relations, and stress management [15]. This scale has been translated into different languages and was tested under various cultural backgrounds, and the results all showed consistently good reliability and validity. For example, Cronbach coefficient of the Iranian version, the Kurdish version, and the Japanese version were all above 0.8 [16–18]. This scale has also been tested among different populations, including college students, elderly people, middle-aged women, chronic disease residents and etc. [13]. In China, it was firstly translated into Chinese and tested among Taiwanese people [19], and later on it was also used among individuals in mainland [20]. However, previous studies, yet limited, were all focusing on special populations, such as elderly residents with chronic disease or college students in the local healthcare system [21, 22]. In the meantime, the previous studies were done under the past “medical oriented” healthcare system environment. Ever since 2017, the Chinese government began to build an integrated healthcare system to promote the healthy lifestyle among local residents. Some strategies, such as physical and nutrition prescription, early detection screen for chronic disease and related health education, set up self-service health rooms, health theme parks, and etc., had been implemented [23, 24]. As such, it would be meaningful to measure the current HPLP level among general residents in China under the integrated healthcare system, and this might be helpful for the future public health planning for all citizens.

HPLP level might be affected by a lot of factors, such as gender, age, income, education, employment status, lifestyles of family members [25–27]. However, these factors might vary due to culture differences. For example, in Japan a study among college students found that being female, at relatively senior levels, and living with family were positively associated with HPLP-II scores [18]. In comparison, another study among Iranian elderlies found that being male, age below 75 years old, and with higher education level were significantly associated with higher scores of HPLP-II [25].

Previous studies on HPLP tended to focus more on its relationship with individuals’ self-perceived health status, while less attention was given to how it is related with individuals’ health management intention and behaviors. For example, one study among adult cancer survivors in USA found that those with better perceived health status showed much higher level HPLP scores [28]. Another study found that all the HPLP domain scores were positively associated with self-rated health status, and negatively with self-reported health complaints in urban Chinese women [29]. However, it is important to explore how HPLP might affect individuals’ health intention and behaviors as this might help to understand how it affects individuals’ health in general.

The current study aims to 1) evaluate the HPLP levels among general citizens in China under the integrated healthcare system; 2) explore the significant correlates of HPLP-II total and domain scores; 3) investigate the relationships between health promoting lifestyle with health management intention and behaviors.

## Methods

### Study design & setting

A cross-sectional study was carried out from July to August 2019. Data were collected using self-administered questionnaires. Three cities (Hangzhou, Deqing, and Yuhuan) were selected to represent the well-, middle-, and less-developed economy levels in Zhejiang Province, and each city has two established integrated health service groups. One hospital and two of its associated community health centers were chosen from each health service groups from each city. In total, six county hospitals and 12 community health centers were selected as the investigation sites, the name of the participating health organizations were listed in the S1 File.

### Participants

Residents waiting in the outpatient hall of each hospital/community health centers were approached. Participants had to meet the following eligibility criteria:1) aged 18 to 80 years; 2) lived in the local area for more than half a year; 3) had used local health services; 4) without any cognitive disability. Recommendations on sample size for studies using scales suggested a minimum of 10 subjects per item are needed [30]. Since HPLP-II scale has 52 items, the minimum sample size should be 520. In our study, 743 residents completed the survey in the end. After removing those with missing items in the survey, the final sample was 666.

### Study measures

Health promoting lifestyle profile-II (HPLP-II) has 52 items, and it was revised from an initial version with 48 items which was developed back in 1987 [13]. It has been translated from English to Chinese with cultural validations. It has a good internal consistency (Cronbach’s alpha 0.91) [31]. The Chinese version HPLP-II comprises of the same 52 items, covering six domains: health responsibility (9 items, e.g. ask for information from health professionals about how to take good care of myself, read or watch TV program about improving health, attend educational programs on personal health care, report any unusual signs or symptoms to a physician or other health professional etc.), physical activity (8 items, e.g. follow a planned exercise program, exercise vigorously for 20 or more minutes at least three times a week, do stretching exercise at least 3 times per week, reach my target heart rate when exercising etc.), nutrition (9 items, e.g. choose a diet low in fat, saturated fat, and cholesterol, limit use of sugars and food containing sugar, eat breakfast, read labels to identify nutrients etc.), spiritual growth (9 items, e.g. feel I am growing and changing in positive ways, believe that my life has purpose, look forward to the future, am aware of what is important to me in life, etc.), interpersonal relations (9 items, e.g. discuss my problems and concerns with people close to me, praise other people easily for their achievements, spend time with close friends, settle conflicts with others through discussion and compromise etc.), stress management (8 items, e.g. get enough sleep, take some time for relaxation each day, concentrate on pleasant thoughts at bedtime, use specific methods to control my stress etc.). In the current study, the participants were required to rate on a 4-point Likert scale from 1 (not at all) to 4 (always). All item scores were summed up, with the total score of HPLP-II ranged between 52 and 208. Each domain can be used independently, domain scores of physical activity and stress management ranged between 8 and 32, and the other four domains ranged from 9 to 36. Higher scores indicated healthier lifestyles [32]. According to the previous study, a total HPLP-II score between 52 and 104 is considered as poor, between 105 and 156 as moderate, and between 157 and 208 as good [21]. In this study, the Cronbach’s α of the overall scale is 0.95. The Cronbach’s α of each domain is: health responsibility (0.81), physical activity (0.83), nutrition (0.72), spiritual growth (0.84), interpersonal relations (0.84), stress management (0.80).

Health management intentions and behaviors were measured by six self-developed questions, with three on intentions and three on behaviors, the questions list could be found in the S2 File.

### Statistical analysis

Data were entered through EpiData 3.1 (The Epidata Association, Odense, Denmark) and analyzed using SPSS 23.0 (SPSS Inc., Chicago, IL, USA). Descriptive analyses were performed for socio-demographic variables. Categorical data were listed as frequency and percentage, while age as the only continuous socio-demographic variable was shown as mean and standard deviation (SD). HPLP-II scale scores were shown as mean and standard deviation. Separate multiple linear regressions were conducted to explore the significant correlates of all six domains of HPLP-II, with each being the dependent variable and regressed on gender, age, occupation, education level, monthly per capita income. Binary logistic regressions were conducted to explore the significant correlates of each health management intentions and behaviors separately, with six domanial scores of HPLP-II being the independent variables after controlling for socio-demographic variables. The significant level of all the regressions were set as P<0.05.

### Ethics considerations

This study was approved by the Hangzhou Normal University Ethics Board (University of Zhejiang, China). Written informed consent form was obtained from each participant prior to the enrolment. A copy of the signed consent form was given to each participant.

## Results

### Sample characteristics

Table 1 shows the socio-demographic characteristics of the study sample. The mean age of the subjects was 38.7 years old (SD = 15.6), 59.2% of them were female, and 41.7% were self-employed. A majority of the subjects (78.3%) had an education level of Junior college and below. More than half of the subjects (61.7%) have a monthly per capita income of 5000 RMB or less.

**Table 1:** Sample characteristics [n(%)] (N = 666).

Variate	$n/\bar{x}$	%/SD
**Gender**		
Male	272	40.8
Female	394	59.2
**Age**	38.7	15.6
<30	225	33.8
30–39	159	23.8
40–49	124	18.6
50–59	79	11.9
≥60	79	11.9
**Occupation**		
Self-employed	278	41.7
White collar	171	25.7
Blue collar	122	18.3
Student	56	8.4
Unemployed	39	5.9
**Education level**		
primary school and below	89	13.4
Middle school	164	24.6
High school	139	20.9
Junior college	129	19.4
undergraduate college and above	145	21.7
**Monthly Per Capita Income**		
less than 3000 RMB	156	23.4
3001–5000 RMB	255	38.3
5001–8000 RMB	149	22.4
more than 8000 RMB	106	15.9

### The mean scores of HPLP-II

The average and total scores of each HPLP-II domain and the overall scale are listed in Table 2. The average total score of HPLP-II was 130.02±23.19. For domain scores, interpersonal relationship (2.68±0.50) had the highest average score, while physical activity (2.21±0.59) had the lowest average score.

**Table 2:** HPLP-II domain and total scores.

Variate	Items	Scores	Average score of each domain ($\bar{x}\pm \mathrm{SD}$)	Average score of each item ($\bar{x}\pm \mathrm{SD}$)
**Health responsibility**	9	9~36	22.21±4.60	2.47±0.51
**Physical activity**	8	8~32	17.68±4.73	2.21±0.59
**Nutrition**	9	9~36	23.24±4.26	2.58±0.47
**Spiritual growth**	9	9~36	22.89±4.84	2.54±0.54
**Interpersonal relations**	9	9~36	24.16±4.53	2.68±0.50
**Stress management**	8	8~32	19.84±4.07	2.48±0.51
**Total scores**	52	52~208	130.02±23.19	2.50±0.45

### The influence of socio-demographic characteristics on HPLP-II scale scores

The association between socio-demographic characteristics and health promoting lifestyles were analyzed by liner regression, and shown in Table 3. Score of HPLP-II was negatively associated with being male (β = -0.13, p<0.01; Ref: female), positively associated with being students (β = 0.15, p<0.01; Ref: self-employed) and with a monthly per capita income of 8000 RMB or above (β = 0.15, p<0.01; Ref: less than 3000 RMB).

**Table 3:** Liner regression of demographic characteristics on each domain scores of HPLP-II scale (Standardizedβvalue).

Variables	Health responsibility	Physical activity	Nutrition	Spiritual growth	Interpersonal relations	Stress management	Total scores
**Gender**							
Female	1	1	1	1	1	1	1
Male	-0.15***	-0.06	-0.22***	-0.09*	-0.09*	-0.06	-0.13**
**Age**	0.12*	0.06	0.11*	0.02	0.10	0.14*	0.10
**Occupation**							
Self-employed	1	1	1	1	1	1	1
White collar	0.07	0.06	0.05	-0.01	0.03	0.03	0.04
Blue collar	-0.02	0.02	0.01	0.01	-0.04	0.01	-0.01
Student	0.06	0.15**	0.09*	0.13**	0.14**	0.19***	0.15**
Unemployed	-0.05	-0.06	-0.01	-0.11**	-0.05	-0.03	-0.06
**Education level**							
primary school and below	1	1	1	1	1	1	1
Middle school	0.01	0.06	0.01	-0.02	-0.03	0.03	0.01
High school	-0.01	0.04	-0.08	-0.01	-0.02	-0.03	-0.02
Junior college	0.10	0.10	-0.07	0.01	0.02	0.02	0.04
undergraduate college and above	0.09	0.10	-0.02	0.01	0.06	0.06	0.06
**Monthly Per Capita Income**							
less than 3000 RMB	1	1	1	1	1	1	1
3001–5000 RMB	0.01	0.03	-0.01	0.01	0.07	0.03	0.03
5001–8000 RMB	0.04	0.02	0.03	0.05	0.07	0.04	0.05
more than 8000 RMB	0.10	0.07	0.12*	0.17***	0.19***	0.12*	0.15**

*P<0.05

**P<0.01

***P<0.001.

For domain scores, health responsibility was positively associated with age (β = 0.12, p<0.05), and negatively associated with being male (β = -0.15, p<0.001; Ref: female). Physical activity was positively associated with being students (β = 0.15, p<0.01; Ref: self-employed). Nutrition was negatively associated with being male (β = -0.22, p<0.001; Ref: female), and positively associated with age (β = 0.11, p<0.05), being students (β = 0.09, p<0.05; Ref: self-employed), with a monthly per capita income of 8000 RMB or above (β = 0.12, p<0.05; Ref: less than 3000 RMB). Spiritual growth was negatively associated with male (β = -0.09, p<0.05; Ref: female), unemployed (β = -0.11, p<0.01; Ref: self-employed), and positively associated with student (β = 0.13, p<0.01; Ref: self-employed), with a monthly per capita income of 8000 RMB or above (β = 0.17, p<0.001; Ref: less than 3000 RMB). Interpersonal relations were negatively associated with being male (β = -0.09, p<0.05; Ref: female), positively associated with being student (β = 0.14, p<0.01; Ref: self-employed) and with a monthly per capita income of 8000 RMB or above (β = 0.19, p<0.001; Ref: less than 3000 RMB). Stress management score were positively associated with age (β = 0.14, p<0.05), being students (β = 0.19, p<0.001; Ref: self-employed), and with a monthly per capita income of 8000 RMB or above (β = 0.12, p<0.05; Ref: less than 3000 RMB).

### The influence of HPLP-II scale scores on health management intentions and behaviors

The association between health promoting lifestyles and health management intentions/behaviors were analyzed by binary logistic regression, and shown in Table 4. Perceived good health status was positively associated with spiritual growth (OR = 1.13,95%CI:1.05~1.21). Intention of regular medical examinations was positively associated with health responsibility (OR = 1.14, 95%CI:1.07~1.22), physical activity (OR = 1.07,95%CI:1.01~1.13), interpersonal relations (OR = 1.09, 95%CI:1.01~1.17), and negatively associated with stress management (OR = 0.86, 95%CI:0.80~0.93). Increasing demand of community health managers was positively associated with health responsibility (OR = 1.08, 95%CI:1.01~1.17), and negatively associated with interpersonal relations (OR = 0.91, 95%CI:0.84~0.99). Following doctors’ advice was positively associated with health responsibility (OR = 1.08, 95%CI:1.01~1.14), nutrition (OR = 1.07, 95%CI:1.01~1.14), and interpersonal relations (OR = 1.08, 95%CI:1.00~1.16). Having a family doctor and choosing community health institutions as the initial medical treatment have no association with any domains of HPLP-II.

**Table 4:** Binary logistic regression of HPLP-II scale scores on health management intentions and behaviors [OR(95%CI)].

Variables	Perceived good health status N = 654	Intention of regular medical examinations N = 665	Increasing demand of community health managers N = 653	Follow doctors’ advice N = 661	Have a family doctor N = 618	Choose community health service institutions as the initial medical treatment N = 656
**Health responsibility**	0.97(0.91,1.03)	1.14(1.07,1.22)***	1.08(1.01,1.17)*	1.08(1.01,1.14)*	1.05(0.98,1.14)	1.06(0.99,1.13)
**Physical activity**	1.02(0.97,1.08)	1.07(1.01,1.13)*	1.03(0.97,1.10)	0.95(0.89,1.00)	1.05(0.98,1.12)	1.03(0.97,1.09)
**Nutrition**	1.02(0.96,1.09)	1.03(0.97,1.10)	1.02(0.94,1.09)	1.07(1.01,1.14)*	1.03(0.96,1.11)	0.99(0.93,1.05)
**Spiritual growth**	1.13(1.05,1.21)***	0.95(0.88,1.01)	1.04(0.96,1.12)	0.96(0.90,1.03)	0.99(0.91,1.08)	0.99(0.92,1.06)
**Interpersonal relations**	0.93(0.87,1.00)	1.09(1.01,1.17)*	0.91(0.84,0.99)*	1.08(1.00,1.16)*	0.94(0.86,1.03)	0.97(0.90,1.04)
**Stress management**	1.03(0.95,1.11)	0.86(0.80,0.93)***	1.00(0.91,1.09)	1.03(0.95,1.11)	1.01(0.92,1.12)	0.95(0.88,1.03)

*P<0.05

**P<0.01

***P<0.001.

Adjusted for gender, age, education level, occupation, monthly per capita income.

## Discussion

This is the first large scale survey to assess the healthy lifestyle levels of community residents in China under the new integrated healthcare system. The total score of HPLP-II among community residents in Zhejiang Province is 130.02±23.19, which is at a moderate level. Compare with other countries, the HPLP total score was very close to that of USA and Japan in 2011 [33]. Among the six subscales of HPLP-II, the highest subscale score is interpersonal relations, and the lowest subscale score is physical activity. This is similar with the results from other countries [26, 34–36].

Findings from the current study suggested that factors influencing the HPLP-II scores included gender, age, occupation, and family income. These factors are consistent with findings from other studies [25, 26, 34]. But for each domain, their influencing factors might vary depending on where the studies were conducted. Among our sample, male residents were found to report significant lower scores of health responsibility, nutrition, spiritual growth, and interpersonal relations. This is very similar to findings from the Japanese study [33]. One possible explanation is that both countries shared similar Asian family culture of ‘male leads outside while female leads inside’, and such belief is still quite common in China nowadays. As a result, females usually need to pay more attention on family members’ health and nutrition [37]. Spiritual growth is about individual’s positivity towards future. Compared to male residents, females tend to be more optimistic [38]. One possibility is that men usually face greater pressures such as from work or higher expectation from the society [39, 40]. Good interpersonal relations indicated good social support from friends. Compare to men, women are more willing to seeking emotional support from friends as this is one of their coping mechanisms to relieve their stress [41]. As such, female residents generally scored higher on interpersonal relations.

Residents at older age group were found to report significantly higher level of health responsibility, nutrition, and stress management compared to those in the younger age groups. This could be partially due to the fact that as individuals getting older, they are more likely to face health problems and thus having higher awareness on health. This result is the same as that found in Japan and North of USA, except on health responsibility [33]. Higher health responsibility among elder residents might be due to the fact that there are very limited numbers of family doctors in China [42, 43], and the service priority was given to elder citizens ever since 2016 as requested by the government. According to the requirements of national family doctor contract services, family doctors should carry out regular health education and follow-up services for the elderlies at their own home, which might cause the increase of their health awareness [44].

Compare to those who were self-employed, being students had significantly higher scores on almost all domains of HPLP-II. For students in China, as health related courses are usually part of the syllabus; in the meantime, they are also more likely to participate in daily sports activities and have close friendship with classmates, as such they are more likely to have healthier lifestyles than the working adults [18]. Compare to self-employed occupation, unemployed people were found negatively associated with spiritual growth. This is similar with other results among unemployed people [45, 46]. Lastly, residents with higher monthly family incoming levels had significantly higher HPLP scores on nutrition, spiritual growth, interpersonal relations and stress management. This is understandable as in most of the cases people with higher incomes tend to pay more attention to their own health and also have the ability to invest more on their health [47, 48].

The logistic regression suggested that most domains of HPLP-II are positively associated with better health management intentions, including perceived good health status, intention of regular medical examination, and increasing demand of community health managers. This is similar to findings from previous studies [29, 35, 49]. However, there are two exceptions: between stress management and intention of regular medical examination, and between interpersonal relations and demand of community health managers. Stress management is related to individuals’ mental health. It reflects individual’s capability of recognizing the stressors, and take measures to control the damage to their health [29]. As mental health and physical health are inter-related, it’s possible that individuals with good mental health status tend to have an overall high rating on their perceived health status and result in lower intention of regular physical examination [50]. Higher scores on interpersonal relations indicates better social supports received from family members or friends. In the meantime, studies also found that maintain good social support might reduce health-care demands and misconceived health beliefs among patients [51].

Regarding health management behaviors, our results suggested that HPLP are only significantly correlated with following doctor’s advice, but not associated with having a family doctor and choosing community health service institutions as the initial medical treatment. A previous qualitative study among Chinese residents found that lower awareness on health responsibility is a barrier to the utilization of family doctor and choose community health center as initial medical treatment [52–54]. However, our study provides a different view as these two seems to be less relevant since having family doctor and utilization of community health service really more depends on individual’s confidence towards them. According to a previous study, the performance of local community health service is still not as good as expected [55]. Also, unlike in western countries where there is a strict hierarchical medical system [56], residents in China can choose the health service providers freely which makes it more difficult for family doctors and community health service centers to compete [57].

### Limitations

There are some limitations of this study. Firstly, this study was only done in Zhejiang Province, thus cannot represent the overall situation in China. Secondly, data were collected through self-administered questionnaire, which might cause recall bias. Thirdly, the current sample was relatively young, which might affect the representativeness of the findings.

## Conclusions

The healthy lifestyle of community residents in Zhejiang Province of China are at moderate levels, and there is still space for further improvements. Most domains of HPLP-II are positively associated with better health management intentions and increasing demand of community health managers, but not with health management behavior on following doctor’s advice. Special attention should be given to residents who are male, at younger and middle age group, being self-employed, and with low-income levels, to improve their healthy lifestyle levels.

## Supporting information

S1 File Participating hospitals/institutions as the investigation sites. (DOCX)

S2 File Self-developed question on health management intentions and behaviors. (DOCX)

S1 Data (SAV)
