# Supplementary material for: Influence of health promoting lifestyle on health management intentions and behaviors among Chinese residents under the integrated healthcare system
Source: PLoS One. 2022 Jan 25;17(1):e0263004. doi: 10.1371/journal.pone.0263004 (PMC8789132; doi:10.1371/journal.pone.0263004)
Supplement: S1 File — (DOCX) [file pone.0263004.s001.docx]

**Participating Hospitals/Institutions as the Investigation Sites**

**Hangzhou City**

Chun’an First People’s Hospital

Chun’an County Hospital of Traditional Chinese Medicine

Qiaodao Lake Community Health Service Center

Weiping Central Community Health Service Center

Fenkou Central Community Health Service Center

Fengshuling Central Community Health Service Center

**Deqing City**

Deqing People’s Hospital

Deqing Hospital

Fuxi Community Health Service Center

Luoshe Community Health Service Center

Xin’an Community Health Service Center

Zhongguan Community Health Service Center

**Yuhuan City**

Yuhuan First People’s Hospital

Yuhuan Second People’s Hospital

Kanmen Community Health Service Center

Damaiyu Central Community Health Service Center

Qinggang Community Health Service Center

Shamen Community Health Service Center
