# Supplementary material for: Influence of health promoting lifestyle on health management intentions and behaviors among Chinese residents under the integrated healthcare system
Source: PLoS One. 2022 Jan 25;17(1):e0263004. doi: 10.1371/journal.pone.0263004 (PMC8789132; doi:10.1371/journal.pone.0263004)
Supplement: S2 File — (DOCX) [file pone.0263004.s002.docx]

**Self-developed Question on Health Management Intentions and Behaviors**

English Version

Questions on intentions:

1、How do you feel about your health status?

2、Do you think it is necessary to have regular medical examinations?

3、Do you feel there is a need to have more community health managers?

Questions on behaviors:

1、Do you usually follow doctors’ advice (e.g., take medicine, diet and exercise)?

2、Do you have a family doctor?

3、Would you choose community health service institutions to have your initial medical treatment？

Chinese Version

Questions on intentions:

1、您觉得您的健康状况如何？

2、您认为有必要定期进行体检吗？

3、您觉得对社区健康管理人员的需求有增加吗？

Questions on behaviors:

1、您遵从医嘱吗（比如用药、饮食运动等指导）？

2、您有签约家庭医生吗？

3、您是否会选择去社区卫生服务机构首诊？
